# Chloridomethyl­phen­yl(quinoline-2-carboxyl­ato-κ^2^ *N*,*O*)tin(IV)

**DOI:** 10.1107/S1600536810027595

**Published:** 2010-07-17

**Authors:** Maryam Vafaee, Mostafa M. Amini, Seik Weng Ng

**Affiliations:** a Department of Chemistry, General Campus, Shahid Beheshti University, Tehran 1983963113, Iran; b Department of Chemistry, University of Malaya, 50603 Kuala Lumpur, Malaysia

## Abstract

The Sn atom in the title compound, [Sn(CH_3_)(C_6_H_5_)(C_10_H_6_NO_2_)Cl], shows a distorted C_2_SnNOCl trigonal-bipyramidal coordination; the apical sites are occupied by the N and Cl atoms.

## Related literature

For chloridodimeth­yl(quinoline-2-carboxyl­ato)tin(IV), see: Wang *et al.* (2007 ▶).
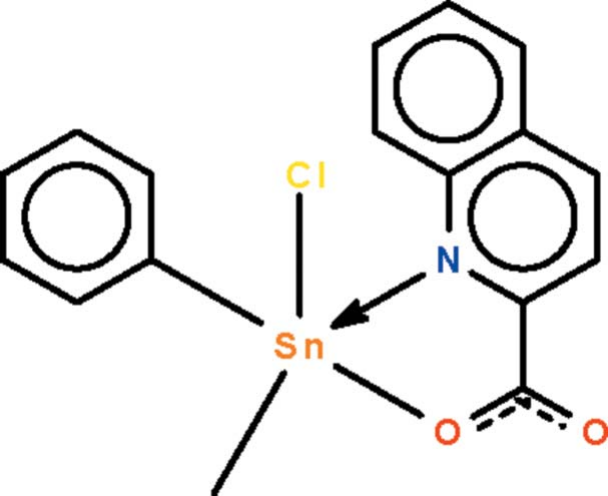


## Experimental

### 

#### Crystal data

[Sn(CH_3_)(C_6_H_5_)(C_10_H_6_NO_2_)Cl]
*M* *_r_* = 418.43
Monoclinic, 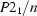

*a* = 10.6942 (5) Å
*b* = 13.1463 (6) Å
*c* = 11.2733 (5) Å
β = 93.689 (1)°
*V* = 1581.6 (1) Å^3^
*Z* = 4
Mo *K*α radiation
μ = 1.79 mm^−1^
*T* = 100 K
0.30 × 0.20 × 0.10 mm

#### Data collection

Bruker SMART APEX diffractometer
Absorption correction: multi-scan (*SADABS*; Sheldrick, 1996 ▶) *T*_min_ = 0.616, *T*_max_ = 0.841
14187 measured reflections
3613 independent reflections
3433 reflections with *I* > 2σ(*I*)
*R*_int_ = 0.039

#### Refinement

*R*[*F* ^2^ > 2σ(*F* ^2^)] = 0.035
*wR*(*F* ^2^) = 0.096
*S* = 1.40
3613 reflections
201 parameters
H-atom parameters constrained
Δρ_max_ = 1.74 e Å^−3^
Δρ_min_ = −0.94 e Å^−3^

### 

Data collection: *APEX2* (Bruker, 2009 ▶); cell refinement: *SAINT* (Bruker, 2009 ▶); data reduction: *SAINT*; program(s) used to solve structure: *SHELXS97* (Sheldrick, 2008 ▶); program(s) used to refine structure: *SHELXL97* (Sheldrick, 2008 ▶); molecular graphics: *X-SEED* (Barbour, 2001 ▶); software used to prepare material for publication: *publCIF* (Westrip, 2010 ▶).

## Supplementary Material

Crystal structure: contains datablocks global, I. DOI: 10.1107/S1600536810027595/bt5296sup1.cif

Structure factors: contains datablocks I. DOI: 10.1107/S1600536810027595/bt5296Isup2.hkl

Additional supplementary materials:  crystallographic information; 3D view; checkCIF report

## Figures and Tables

**Table:** 

Sn1—C1	2.117 (3)
Sn1—C2	2.122 (2)
Sn1—N1	2.369 (2)
Sn1—O1	2.062 (2)
Sn1—Cl1	2.4414 (7)

**Table:** 

C1—Sn1—C2	138.58 (9)

**Fig. 1.**
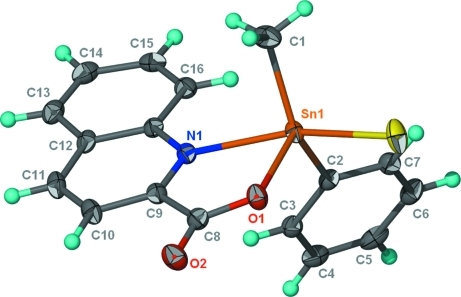
Anisotropic displacement ellipsoid plot (Barbour, 2001) of SnCl(CH3)(C6H5)(C10H6NO2) at the 70% probability level. Hydrogen atoms are drawn as spheres of arbitrary radius.

## supplementary crystallographic information

### Comment

The tin atom in chlorodimethyl(quinoline-2-carboxylate)tin shows *cis*-C_2_SnNOCl trigonal bipyramidal coordination, with the C_2_Sn skeleton being bent at 131.8 (2) ° (Wang *et al.*, 2007). Replacing one of the methyl groups by a bulkier phenyl group in the title analog (Scheme I, Fig. 1) leads to only a marginal opening of the angle 138.6 (1) °; the *trans* angle is also marginally increased.

### Experimental

Sodium quinoline-2-carboxylate was synthesized by reacting sodium hydroxide and quinoline-2-carboxylic acid in toluene in a Dean-Start apparatus. The compound (0.20 g, 1 mmol) was then stirred with methylphenyltin dichloride (0.35 g, 1 mmol) in toluene; no heat was applied. The precipitate that formed was recrystallized from methanol.

### Refinement

Hydrogen atoms were placed in calculated positions (C–H 0.95–0.98 Å) and were included in the refinement in the riding model approximation, with *U*(H) set to 1.2–1.5*U*_eq_(C).

The final difference Fourier map had a peak (1.741eÅ^-3^) at 0.78 Å from Sn1.

### Crystal data

**Table:**

[Sn(CH_3_)(C_6_H_5_)(C_10_H_6_NO_2_)Cl]	*F*(000) = 824
*M**_r_* = 418.43	*D*_x_ = 1.757 Mg m^−^^3^
Monoclinic, *P*2_1_/*n*	Mo *K*α radiation, λ = 0.71073 Å
Hall symbol: -P 2yn	Cell parameters from 9943 reflections
*a* = 10.6942 (5) Å	θ = 2.4–28.3°
*b* = 13.1463 (6) Å	µ = 1.79 mm^−^^1^
*c* = 11.2733 (5) Å	*T* = 100 K
β = 93.689 (1)°	Prism, colorless
*V* = 1581.6 (1) Å^3^	0.30 × 0.20 × 0.10 mm
*Z* = 4	

### Data collection

**Table:**

Bruker SMART APEX diffractometer	3613 independent reflections
Radiation source: fine-focus sealed tube	3433 reflections with *I* > 2σ(*I*)
graphite	*R*_int_ = 0.039
ω scans	θ_max_ = 27.5°, θ_min_ = 2.4°
Absorption correction: multi-scan (*SADABS*; Sheldrick, 1996)	*h* = −13→12
*T*_min_ = 0.616, *T*_max_ = 0.841	*k* = −17→17
14187 measured reflections	*l* = −14→14

### Refinement

**Table:**

Refinement on *F*^2^	Secondary atom site location: difference Fourier map
Least-squares matrix: full	Hydrogen site location: inferred from neighbouring sites
*R*[*F*^2^ > 2σ(*F*^2^)] = 0.035	H-atom parameters constrained
*wR*(*F*^2^) = 0.096	*w* = 1/[σ^2^(*F*_o_^2^) + (0.0523*P*)^2^ + 0.1663*P*] where *P* = (*F*_o_^2^ + 2*F*_c_^2^)/3
*S* = 1.40	(Δ/σ)_max_ = 0.001
3613 reflections	Δρ_max_ = 1.74 e Å^−^^3^
201 parameters	Δρ_min_ = −0.94 e Å^−^^3^
0 restraints	Extinction correction: *SHELXL97* (Sheldrick, 2008), Fc^*^=kFc[1+0.001xFc^2^λ^3^/sin(2θ)]^-1/4^
Primary atom site location: structure-invariant direct methods	Extinction coefficient: 0.0280 (15)

### Fractional atomic coordinates and isotropic or equivalent isotropic displacement parameters (Å^2^)

**Table:**

	*x*	*y*	*z*	*U*_iso_*/*U*_eq_	
Sn1	0.813061 (14)	0.776948 (11)	0.733408 (14)	0.01396 (11)	
Cl1	1.02998 (6)	0.75394 (6)	0.68330 (7)	0.02452 (16)	
O1	0.77940 (15)	0.62981 (12)	0.67664 (15)	0.0176 (3)	
O2	0.64876 (17)	0.49885 (13)	0.64587 (16)	0.0217 (4)	
C1	0.8465 (3)	0.77556 (17)	0.9205 (3)	0.0226 (6)	
H1A	0.7742	0.8046	0.9575	0.034*	
H1B	0.9213	0.8160	0.9428	0.034*	
H1C	0.8596	0.7054	0.9479	0.034*	
C2	0.7644 (2)	0.88296 (16)	0.59620 (19)	0.0139 (4)	
C3	0.6502 (2)	0.87698 (17)	0.52921 (19)	0.0161 (4)	
H3	0.5931	0.8236	0.5435	0.019*	
C4	0.6195 (2)	0.94912 (19)	0.4413 (2)	0.0193 (5)	
H4	0.5427	0.9438	0.3945	0.023*	
C5	0.7010 (2)	1.02855 (18)	0.4224 (2)	0.0203 (5)	
H5	0.6785	1.0791	0.3647	0.024*	
C6	0.8157 (2)	1.03451 (17)	0.4877 (2)	0.0197 (5)	
H6	0.8719	1.0887	0.4742	0.024*	
C7	0.8479 (2)	0.96066 (17)	0.5731 (2)	0.0167 (4)	
H7	0.9274	0.9634	0.6158	0.020*	
C8	0.6698 (2)	0.58635 (18)	0.6779 (2)	0.0163 (4)	
C9	0.5661 (2)	0.65049 (17)	0.72434 (19)	0.0146 (4)	
C10	0.4453 (2)	0.61082 (18)	0.7341 (2)	0.0178 (4)	
H10	0.4255	0.5435	0.7089	0.021*	
C11	0.3567 (2)	0.67171 (19)	0.7810 (2)	0.0187 (5)	
H11	0.2747	0.6462	0.7898	0.022*	
C12	0.3874 (3)	0.77218 (16)	0.8162 (2)	0.0158 (5)	
C13	0.3008 (2)	0.83899 (19)	0.8662 (2)	0.0193 (5)	
H13	0.2194	0.8153	0.8813	0.023*	
C14	0.3339 (2)	0.9378 (2)	0.8929 (2)	0.0207 (5)	
H14	0.2759	0.9819	0.9271	0.025*	
C15	0.4546 (2)	0.97368 (18)	0.86940 (19)	0.0186 (5)	
H15	0.4754	1.0429	0.8845	0.022*	
C16	0.5418 (2)	0.91036 (18)	0.82531 (19)	0.0172 (4)	
H16	0.6234	0.9351	0.8126	0.021*	
C17	0.5105 (2)	0.80846 (18)	0.79870 (19)	0.0140 (4)	
N1	0.5977 (2)	0.74461 (18)	0.75477 (18)	0.0139 (4)	

### Atomic displacement parameters (Å^2^)

**Table:**

	*U*^11^	*U*^22^	*U*^33^	*U*^12^	*U*^13^	*U*^23^
Sn1	0.01151 (15)	0.01373 (15)	0.01635 (15)	−0.00098 (4)	−0.00142 (8)	0.00397 (4)
Cl1	0.0122 (3)	0.0219 (3)	0.0395 (4)	0.0004 (3)	0.0017 (2)	0.0078 (3)
O1	0.0146 (8)	0.0141 (8)	0.0244 (9)	−0.0003 (6)	0.0028 (6)	−0.0009 (6)
O2	0.0208 (9)	0.0156 (8)	0.0286 (9)	−0.0014 (7)	0.0008 (7)	−0.0046 (7)
C1	0.0274 (15)	0.0217 (13)	0.0179 (13)	−0.0011 (9)	−0.0052 (11)	0.0035 (8)
C2	0.0152 (10)	0.0134 (10)	0.0133 (9)	0.0019 (8)	0.0027 (8)	0.0011 (8)
C3	0.0148 (11)	0.0176 (11)	0.0162 (10)	0.0009 (8)	0.0020 (8)	−0.0006 (8)
C4	0.0192 (11)	0.0236 (12)	0.0152 (10)	0.0072 (9)	0.0004 (8)	−0.0009 (9)
C5	0.0302 (13)	0.0177 (12)	0.0134 (10)	0.0090 (10)	0.0054 (9)	0.0024 (8)
C6	0.0268 (12)	0.0135 (10)	0.0198 (11)	0.0006 (9)	0.0088 (9)	0.0020 (8)
C7	0.0179 (11)	0.0145 (10)	0.0178 (10)	−0.0005 (8)	0.0021 (8)	−0.0007 (8)
C8	0.0174 (11)	0.0164 (11)	0.0151 (10)	−0.0006 (8)	0.0011 (8)	0.0020 (8)
C9	0.0139 (11)	0.0154 (11)	0.0142 (10)	−0.0005 (8)	−0.0007 (8)	0.0022 (8)
C10	0.0166 (11)	0.0143 (10)	0.0223 (11)	−0.0033 (8)	−0.0002 (9)	0.0030 (8)
C11	0.0142 (11)	0.0196 (12)	0.0223 (11)	−0.0028 (9)	0.0013 (9)	0.0067 (9)
C12	0.0133 (12)	0.0207 (13)	0.0135 (11)	0.0010 (8)	0.0012 (9)	0.0060 (7)
C13	0.0170 (11)	0.0253 (12)	0.0157 (10)	0.0016 (9)	0.0022 (8)	0.0041 (8)
C14	0.0218 (12)	0.0257 (13)	0.0146 (10)	0.0059 (10)	0.0011 (9)	0.0002 (9)
C15	0.0245 (12)	0.0174 (11)	0.0135 (10)	0.0005 (9)	−0.0023 (9)	−0.0012 (8)
C16	0.0193 (11)	0.0176 (11)	0.0147 (10)	−0.0016 (8)	−0.0002 (8)	0.0017 (8)
C17	0.0144 (11)	0.0163 (11)	0.0113 (9)	−0.0004 (9)	0.0001 (8)	0.0029 (8)
N1	0.0136 (10)	0.0142 (9)	0.0139 (9)	−0.0009 (8)	0.0001 (7)	0.0025 (8)

### Geometric parameters (Å, °)

**Table:**

Sn1—C1	2.117 (3)	C7—H7	0.9500
Sn1—C2	2.122 (2)	C8—C9	1.513 (3)
Sn1—N1	2.369 (2)	C9—N1	1.322 (3)
Sn1—O1	2.062 (2)	C9—C10	1.404 (3)
Sn1—Cl1	2.4414 (7)	C10—C11	1.373 (3)
O1—C8	1.305 (3)	C10—H10	0.9500
O2—C8	1.222 (3)	C11—C12	1.412 (4)
C1—H1A	0.9800	C11—H11	0.9500
C1—H1B	0.9800	C12—C13	1.420 (3)
C1—H1C	0.9800	C12—C17	1.426 (3)
C2—C7	1.392 (3)	C13—C14	1.375 (4)
C2—C3	1.397 (3)	C13—H13	0.9500
C3—C4	1.395 (3)	C14—C15	1.415 (4)
C3—H3	0.9500	C14—H14	0.9500
C4—C5	1.385 (4)	C15—C16	1.367 (3)
C4—H4	0.9500	C15—H15	0.9500
C5—C6	1.392 (4)	C16—C17	1.408 (3)
C5—H5	0.9500	C16—H16	0.9500
C6—C7	1.394 (3)	C17—N1	1.371 (3)
C6—H6	0.9500		
			
O1—Sn1—C1	108.43 (8)	C6—C7—H7	119.8
O1—Sn1—C2	111.11 (7)	O2—C8—O1	123.9 (2)
C1—Sn1—C2	138.58 (9)	O2—C8—C9	120.1 (2)
O1—Sn1—N1	73.25 (7)	O1—C8—C9	116.0 (2)
C1—Sn1—N1	90.02 (10)	N1—C9—C10	123.3 (2)
C2—Sn1—N1	89.86 (8)	N1—C9—C8	115.5 (2)
O1—Sn1—Cl1	87.79 (5)	C10—C9—C8	121.2 (2)
C1—Sn1—Cl1	97.31 (9)	C11—C10—C9	118.4 (2)
C2—Sn1—Cl1	96.02 (6)	C11—C10—H10	120.8
N1—Sn1—Cl1	161.00 (7)	C9—C10—H10	120.8
C8—O1—Sn1	123.13 (14)	C10—C11—C12	120.0 (2)
Sn1—C1—H1A	109.5	C10—C11—H11	120.0
Sn1—C1—H1B	109.5	C12—C11—H11	120.0
H1A—C1—H1B	109.5	C11—C12—C13	123.0 (2)
Sn1—C1—H1C	109.5	C11—C12—C17	118.3 (2)
H1A—C1—H1C	109.5	C13—C12—C17	118.7 (2)
H1B—C1—H1C	109.5	C14—C13—C12	120.4 (2)
C7—C2—C3	119.3 (2)	C14—C13—H13	119.8
C7—C2—Sn1	119.21 (16)	C12—C13—H13	119.8
C3—C2—Sn1	121.48 (16)	C13—C14—C15	120.0 (2)
C4—C3—C2	120.2 (2)	C13—C14—H14	120.0
C4—C3—H3	119.9	C15—C14—H14	120.0
C2—C3—H3	119.9	C16—C15—C14	121.1 (2)
C5—C4—C3	120.0 (2)	C16—C15—H15	119.4
C5—C4—H4	120.0	C14—C15—H15	119.4
C3—C4—H4	120.0	C15—C16—C17	119.9 (2)
C4—C5—C6	120.2 (2)	C15—C16—H16	120.1
C4—C5—H5	119.9	C17—C16—H16	120.1
C6—C5—H5	119.9	N1—C17—C16	120.1 (2)
C5—C6—C7	119.7 (2)	N1—C17—C12	120.1 (2)
C5—C6—H6	120.1	C16—C17—C12	119.8 (2)
C7—C6—H6	120.1	C9—N1—C17	119.9 (2)
C2—C7—C6	120.5 (2)	C9—N1—Sn1	112.07 (16)
C2—C7—H7	119.8	C17—N1—Sn1	127.97 (17)
			
C1—Sn1—O1—C8	83.29 (19)	C10—C11—C12—C13	−179.5 (2)
C2—Sn1—O1—C8	−84.14 (18)	C10—C11—C12—C17	1.6 (4)
N1—Sn1—O1—C8	−0.97 (16)	C11—C12—C13—C14	−176.5 (2)
Cl1—Sn1—O1—C8	−179.74 (16)	C17—C12—C13—C14	2.4 (3)
O1—Sn1—C2—C7	−133.04 (16)	C12—C13—C14—C15	0.7 (3)
C1—Sn1—C2—C7	65.2 (2)	C13—C14—C15—C16	−3.0 (3)
N1—Sn1—C2—C7	155.02 (17)	C14—C15—C16—C17	2.2 (3)
Cl1—Sn1—C2—C7	−43.08 (17)	C15—C16—C17—N1	−179.8 (2)
O1—Sn1—C2—C3	47.46 (19)	C15—C16—C17—C12	1.0 (3)
C1—Sn1—C2—C3	−114.4 (2)	C11—C12—C17—N1	−3.5 (3)
N1—Sn1—C2—C3	−24.49 (18)	C13—C12—C17—N1	177.5 (2)
Cl1—Sn1—C2—C3	137.41 (17)	C11—C12—C17—C16	175.8 (2)
C7—C2—C3—C4	−1.0 (3)	C13—C12—C17—C16	−3.2 (3)
Sn1—C2—C3—C4	178.55 (16)	C10—C9—N1—C17	0.2 (3)
C2—C3—C4—C5	−1.7 (3)	C8—C9—N1—C17	−179.51 (19)
C3—C4—C5—C6	2.5 (3)	C10—C9—N1—Sn1	176.98 (17)
C4—C5—C6—C7	−0.5 (3)	C8—C9—N1—Sn1	−2.7 (2)
C3—C2—C7—C6	2.9 (3)	C16—C17—N1—C9	−176.6 (2)
Sn1—C2—C7—C6	−176.62 (17)	C12—C17—N1—C9	2.6 (3)
C5—C6—C7—C2	−2.2 (3)	C16—C17—N1—Sn1	7.2 (3)
Sn1—O1—C8—O2	−179.09 (17)	C12—C17—N1—Sn1	−173.62 (16)
Sn1—O1—C8—C9	−0.1 (3)	O1—Sn1—N1—C9	2.05 (15)
O2—C8—C9—N1	−178.9 (2)	C1—Sn1—N1—C9	−107.22 (17)
O1—C8—C9—N1	2.1 (3)	C2—Sn1—N1—C9	114.19 (16)
O2—C8—C9—C10	1.4 (3)	Cl1—Sn1—N1—C9	5.8 (3)
O1—C8—C9—C10	−177.6 (2)	O1—Sn1—N1—C17	178.5 (2)
N1—C9—C10—C11	−2.1 (3)	C1—Sn1—N1—C17	69.2 (2)
C8—C9—C10—C11	177.6 (2)	C2—Sn1—N1—C17	−69.4 (2)
C9—C10—C11—C12	1.1 (4)	Cl1—Sn1—N1—C17	−177.71 (13)
